# Soil organic carbon pools and carbon management index under different land use systems in North western Himalayas

**DOI:** 10.7717/peerj.15266

**Published:** 2023-06-06

**Authors:** Yasir Hanif Mir, Mumtaz Ahmad Ganie, Tajamul Islam Shah, Aziz Mujtaba Aezum, Shabir Ahmed Bangroo, Shakeel Ahmad Mir, Shahnawaz Rasool Dar, Syed Sheeraz Mahdi, Zahoor Ahmad Baba, Aanisa Manzoor Shah, Uzma Majeed, Tatiana Minkina, Vishnu D. Rajput, Aijaz Ahmad Dar

**Affiliations:** 1 Division of Soil Science and Agricultural Chemistry, Faculty of Agriculture, SKUAST-Kashmir, Wadura, Jammu and Kashmir, India; 2 KVK Shopian, SKUAST-Kashmir, Shopian, Jammu and Kashmir, India; 3 Division of Soil Science, SKUAST-Kashmir, Srinagar, Jammu and Kashmir, India; 4 Research Center for Residue & Quality Analysis, Sheri Kashmir University of Agricultural Sciences & Technologies, Kashmir, Srinagar, Jammu and Kashmir, India; 5 Division of Agronomy, Faculty of Agriculture, SKUAST-Kashmir, Wadura, Jammu and Kashmir, India; 6 Division of Basic Sciences and Humanities, Faculty of Agriculture, SKUAST-Kashmir, Sopore, Jammu and Kashmir, India; 7 Division of Agricultural Statistics, Faculty of Horticulture, SKUAST Kashmir, Srinagar, Jammu and Kashmir, India; 8 Academy of Biology and Biotechnology, Southern Federal University, Oblast, Russia; 9 Directorate of Planning, SKUAST-Kashmir, Srinagar, Jammu and Kashmir, India

**Keywords:** Carbon management index, Soil quality, Soil organic carbon pools, Soil depth, Sustainability

## Abstract

Current study was conducted to evaluate the effect of important land uses and soil depth on soil organic carbon pools viz. total organic carbon, Walkley and black carbon, labile organic carbon, particulate organic carbon, microbial biomass carbon and carbon management index (CMI) in the north Western Himalayas, India. Soil samples from five different land uses viz. forest, pasture, apple, saffron and paddy-oilseed were collected up to a depth of 1 m (0–30, 30–60, 60–90 cm). The results revealed that regardless of soil depth, all the carbon pools differed significantly (*p* < 0.05) among studied land use systems with maximum values observed under forest soils and lowest under paddy-oilseed soils. Further, upon evaluating the impact of soil depth, a significant (*p* < 0.05) decline and variation in all the carbon pools was observed with maximum values recorded in surface (0–30 cm) soils and least in sub-surface (60–90 cm) layers. CMI was higher in forest soils and lowest in paddy-oilseed. From regression analysis, a positive significant association (high R-squared values) between CMI and soil organic carbon pools was also observed at all three depths. Therefore, land use changes and soil depth had a significant impact on soil organic carbon pools and eventually on CMI, which is used as deterioration indicator or soil carbon rehabilitation that influences the universal goal of sustainability in the long run.

## Introduction

SOC is a substantial fertility attribute that affects various soil properties, thus influencing the quality of soil and ecological functions (Benbi et al., 2015; Yadav et al., 2018). It tends to sequester carbon and performs a significant function in the global carbon cycle (Lal, 2018) and climate change (Zhang et al., 2017). However, anthropogenic disturbances have been a significant reason for soil loss and degeneration of carbon stocks, which is a grievous concern to ecosystem sustainability (Yadav et al., 2018). Soils have undergone unabated degradation at a formidable rate owing to wind and water erosion, desertification, and salinization resulting from abuse and inapt agricultural practices. Therefore, it becomes critical to protect them from further deterioration as there is a corresponding depletion in soil quality to produce nutritious crops. Soil organic matter (SOM) is fundamental for agricultural sustainability and its compositional alterations could eventuate in both total and active carbon pools (Blair, Lefroy & Lisle, 1995) where labile fraction includes microbial biomass carbon (MBC), particulate organic matter (POM), easily extractable carbon, readily mineralizable carbon and carbohydrates (Haynes, 2005). Among the fractions, labile organic carbon (LOC) is quite susceptible to the alterations in vegetation and microenvironment of soil and management practices (Sahoo et al., 2019; Cheng, Chen & Luo, 2008; Sainepo, Gachene & Karuma, 2018). Soil carbon is substantially impacted by climate, and land use changes (Lal, 2018), and the transition from natural to the managed system causes SOC pool depletion (Yigini & Panagos, 2016; Somasundaram et al., 2018; Meena et al., 2018). In the Kashmir Himalayas, a significant impact of cropping sequences on carbon sequestration has been observed, resulting in the alteration of soil physical, chemical, and biological properties (Kalambukattu et al., 2013). In addition, conservation tillage and agro-forestry system have been found to have a greater ability for SOC storage among the different management practices in agricultural soils (Babu et al., 2020; Luo, Wang & Sun, 2010; Lal, 2002; Nath & Lal, 2017). Soil stores four times more carbon than the biosphere up to 2 m (Lal, 2001) and fewer alterations in SOC can aid in significant variation in atmospheric carbon dioxide stock (Babu et al., 2020). Thus sustaining SOM is of paramount significance for conserving the quality of soil and ecological balance.

The physical, chemical, and biological properties of soil and the self-organization capacity are directly impacted by organic carbon (OC) pools and carbon lability (Addiscott, 1995; Blair & Crocker, 2000). As a result, their integration into CMI, could serve as a vital metric for assessing the potential of management systems for improving soil quality (Blair, Lefroy & Lisle, 1995; Blair et al., 2006a; Blair et al., 2006b; Diekow et al., 2005). Greenhouse effect could also be reduced by sequestering carbon through suitable land-use systems (Sofi et al., 2016; Han, Li & Horwath, 2013). Forest land use has a maximum potential to sequester soil carbon compared to other land uses (Kooch et al., 2012). However, the prominent land uses of Kashmir valley are under constant stress caused by land conversions owing to cultivation and infrastructural activities (Fayaz et al., 2020), thus reducing carbon sequestration and threatening sustainability. Moreover, most of the studies are concerned with fertility evaluation and crop yields, water, and fertilizer management, however, the impact of LUSs and soil depth on SOC fractions and CMI in the Kashmir Himalayas has not been well studied. Pertaining to the significance of related work, proper comprehension and consideration of research regarding carbon pools and carbon sequestration are needed. Hence, this study was carried out to (i) determine the influence of different LUSs and soil depth on SOC pools, (ii) to evaluate the CMI among LUSs at different depths, and (iii) to identify the relationships between various C fractions and CMI. The outcome is expected to be substantial in order to develop strategies for better soil carbon management and carbon rehabilitation to serve the purpose of global sustainability.

## Materials and Methods

### Study area

A study was carried out across Kashmir Valley in India with an altitude range from 1587–2640 m. Kashmir Valley lies between the coordinates of 33°20′ to 34°54′N and 73°55′ to 75°35′E occupying a total area of 1.5948 million hectares, which includes the net sown area (3.31 lakh hectares), forest (5.24 thousand hectares), permanent pastures (34.92 thousand hectares) and other grazing lands. The soils in the study area are of different groups including hapludalfs, hapludolls, ochraualfs, eutrochrepts, croboralfs, argiudolls, ustifluvents, udifluvents, fluviatile and lacustrine. Five different land uses were selected based on the land use system being followed over the last 30–40 years viz., forest, pasture, apple, saffron, and paddy-oilseed. A purposive random method of sampling was used for the collection of geo-referenced soil samples from preferred LUSs prevailing in the study area. In the forest, soil samples were taken in between the tree rows, in pasture lands, soil samples were taken in the moderately grazed area. However, in apple, soil samples were taken just outside the drip line and in saffron, samples were taken inside corm beds while in the paddy-oilseed cropping system samples were taken inside crop fields. All the samples were collected in the summer season at 0–30, 30–60, and 60–90 cm soil depth. Each soil sample was a composite of four sub-samples, and three soil samples from different locations were collected, for each land use system.

### Physico-chemical properties

Soil samples collected from different locations were air dried, ground and passed through a two mm sieve to analyze different parameters. Particle size distribution was assessed by the hydrometer method in line with Bouyoucos (1962).

Soil reaction (pH) was assessed using a glass electrode pH meter as described by Jackson (1973).

Bulk density (BD) was evaluated by employing the protocols described by Blake & Hartge (1986). Soil cores were taken from each location and from each land use system. The weight of soil + core sampler (Wt_sc_) was recorded and the volume of core sampler (V) was determined, and substituted in the equation:

\begin{eqnarray*}BD= \frac{{\text{Wt}}_{\text{sc}}}{V} \end{eqnarray*}

Available nitrogen (N) was assessed by employing the respective standard method described in Subbiah & Asija (1956), where 5 g soil sample (<2 mm) was treated with 20 ml of 0.32% KMnO_4_, 25 ml of 2.5% sodium hydroxide solution and 10 ml of distilled water followed by distillation. To determine the nitrogen content of the sample, a distillate was collected in a boric acid mixture, and absorbed ammonia was titrated with 0.2 N H_2_SO_4_.

### Soil organic carbon pools

Total organic carbon represents the carbon stocks in the soil which was determined by the wet oxidation as previously described in Snyder & Trofymow (1984). The calculation is as follows:

Mg C trapped = (mean meq H^+^ used in titrating blanks − meq H^+^ used in sample) × 12 mg C/2 meq +

When 1.0 N HCL is used in titrating, this formula reduces to:

Mg C trapped = (ml HCL Blank − ml HCL sample) × 6

Walkley Black carbon (WBC) represents readily oxidizable OC which was evaluated by oxidizing OM using chromic acid, followed by back titration of unconsumed potassium dichromate with ferrous sulfate (Walkley & Black, 1934).

For labile carbon determination, the soil was oxidized with 333 mM KMnO_4_ followed by shaking and centrifugation, and the concentration of KMnO_4_ was quantified at 565 nm wavelength using a spectrophotometer (Blair et al., 1997).

Particulate organic carbon was determined by shaking a suspension (soil + 0.5% sodium hexametaphosphate) for 24 h followed by sieving through 0.053 mm and the retained material was subjected to the wet oxidation method (Camberdella & Elliott, 1992).

MBC was determined using the fumigation extraction method as previously described in Jenkinson & Powlson (1976), where one set was fumigated with fresh ethanol-free chloroform while another set was un-fumigated and extraction with 0.5 M K_2_SO_4_ followed by oxidation with K_2_Cr_2_O_7_ and titration with ferrous ammonium sulfate.

### Carbon management index

CMI is an evaluation methodology that indicates how a certain land type alters the quality of soil in contrast to a reference land use. The CMI was computed from the following relationships:

\begin{eqnarray*}\text{(i) Carbon pool index (CPI)}= \frac{\text{Sample total C}(\mathrm{mg/g})}{\text{Reference total C}(\mathrm{mg/g})} \end{eqnarray*}

(ii) Carbon lability index was calculated as follows:

\begin{eqnarray*}\text{(a) Lability of Carbon}= \frac{\text{C in fraction oxidized by KMnO4}}{\text{C remaining un-oxidized by KMnO4}} \end{eqnarray*}

The lability of carbon was estimated from the difference between labile carbon content and non-labile carbon content (difference between TOC and LOC) of samples.

\begin{eqnarray*}\text{(b) Lability index (LI)}= \frac{\text{Lability of C in sample soil}}{\text{Lability of C in reference soil}} \end{eqnarray*}

(iii) Then, CMI was calculated as follows:

\begin{eqnarray*}\text{CMI}=\text{CPI}\times \text{LI}\times 100 \end{eqnarray*}

CMI was computed for all the land uses; taking native forest soil as a reference with a known CMI of 100.

### Statistical analysis

A two-way ANOVA was employed to evaluate the influence of land use and soil depth on SOC pools. The statistical difference was determined at *P* < 0.05. A correlation coefficient was performed to evaluate the association among physico-chemical characteristics and SOC pools. A simple linear regression was employed to comprehend the relation between CMI and SOC fractions.

## Results and Discussion

### Physico-chemical properties of soils under different land use systems

**Figure 1:**
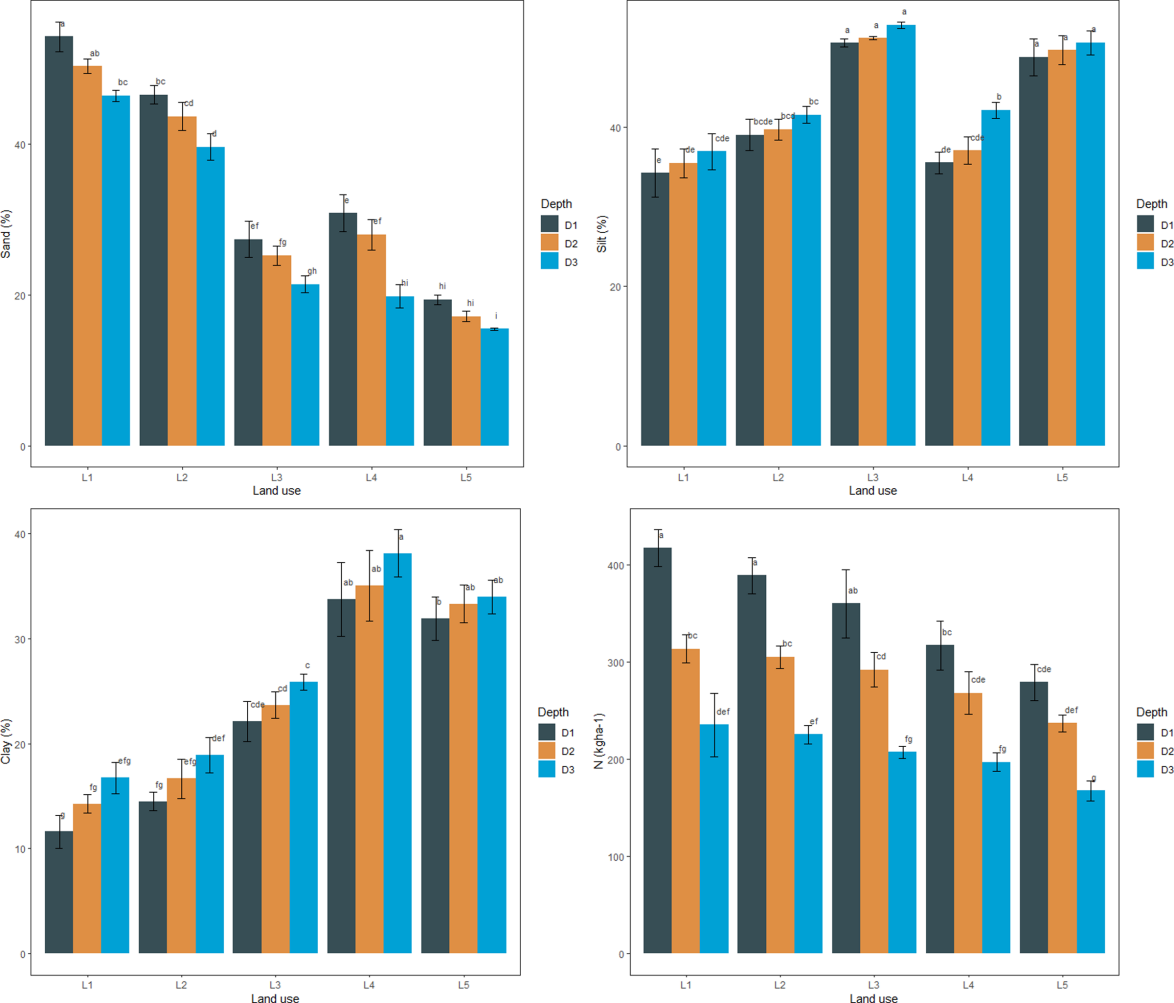
Particle size distribution and nitrogen content under different LUSs and soil depth.

**Figure 2:**
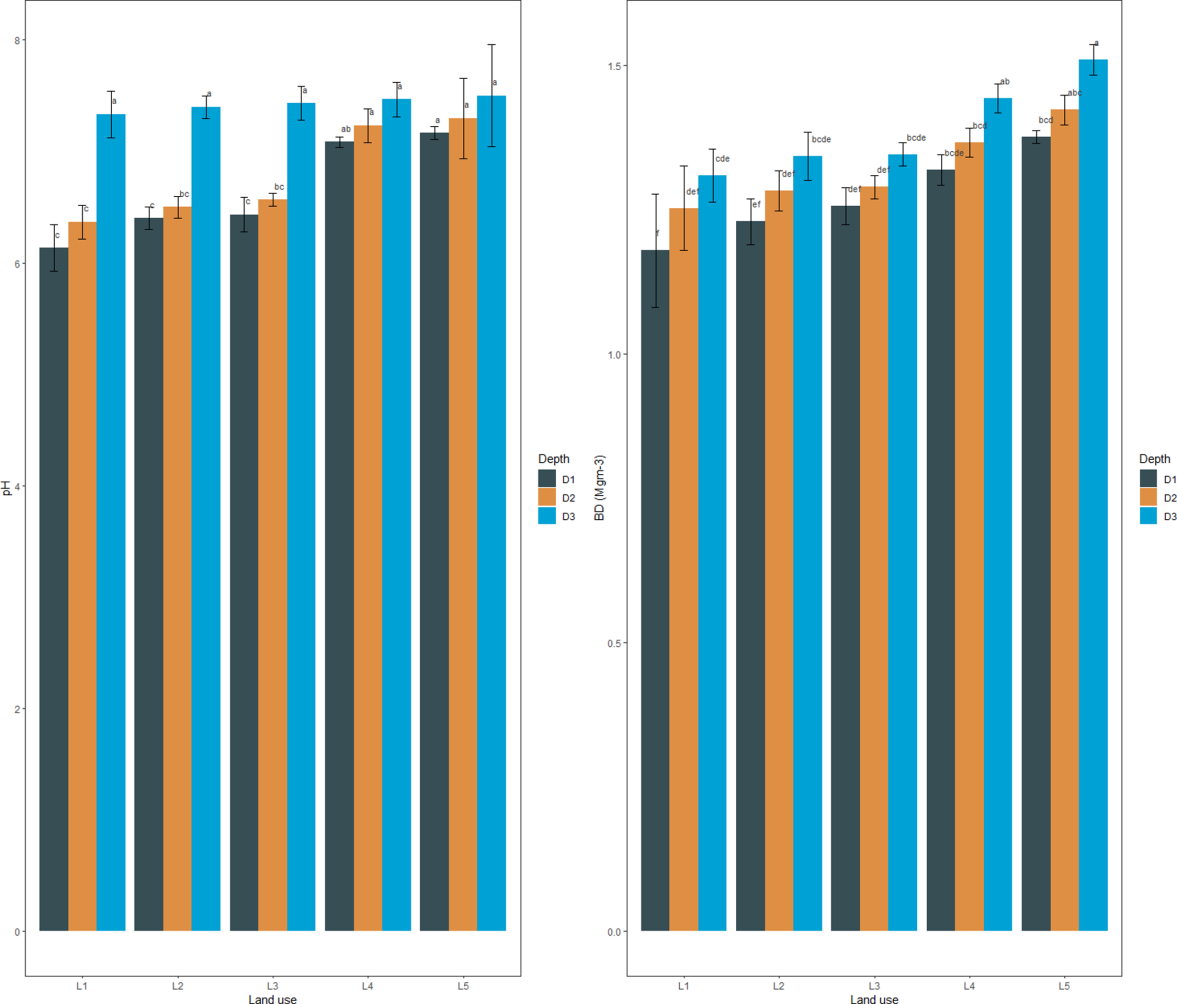
Soil reaction (pH) and bulk density under different LUSs and soil depth.

The mean sand percentage at the surface (0–30 cm) and sub-surface (30–60 and 60–90 cm) soils varied from 19.33–54.20, 17.10–50.30, and 15.46–46.37%, respectively, with the minimum content in paddy-oilseed soils and maximum in forest soils (Fig. 1). The silt percentage at the surface (0–30 cm) and sub-surfaces (30-60 and 60–90 cm) varied from 34.20–50.53, 35.43–51.13, and 36.90–52.76% with the least in paddy-oilseed soils and higher in apple (Fig. 1). The clay percentage varied between 11.60–33.70, 14.26–35.00, and 16.73–38.10% with the maximum in paddy-oilseed soils and minimum in forest soils. The studied land uses were categorized as sandy loam, loam, silt loam, clay loam, and silty clay loam. This is aligned with the reports of Maqbool, Rasool & Ramzan (2017) and Mahapatra et al. (2000). The sand percentage exhibited a declining pattern along the soil depth while silt and clay percentage depicted an increasing pattern along the soil depth, which might be ascribed to illuviation and translocation of clay from upper soil layers (Najar et al., 2009; Mohamed et al., 2017). Forest soils exhibited coarse texture with more percentage of sand and a low percentage of clay owing to steep slopes, reduced soil development, higher altitudes, less infiltration, and vice versa in cultivated soils (Abad, Khosravi & Alamdarlou, 2014; Kiflu & Beyene, 2013; Maqbool, Rasool & Ramzan, 2017). The N levels in surface (0–30 cm) and sub-surfaces (30-60 and 60–90 cm) varied from 279.30–417.52, 237.04–313.85, and 167.67–235.40 kg ha^−1^, respectively, with maximum values in forest soils and minimum in cultivated land uses (Fig. 2). A decline was observed in N levels along the soil depth with a significant (*p* < 0.05) difference across the depths (Table 1, Fig. 1). These findings are in line with the previous reports of scientists (Pal, Panwar & Bhardwaj, 2013; Maqbool, Rasool & Ramzan, 2017). The mean values of BD in surface (0–30 cm) and sub-surface (30-60 and 60–90 cm) soils varied from 1.18–1.38, 1.25–1.42, and 1.31–1.51 Mgm^−3^, respectively, with the minimum values observed under forest soils and maximum under paddy-oilseed soils (Fig. 2). Further, while evaluating the impact of soil depth, an increment was observed and all the examined depths differed significantly (*p* < 0.05) (Table 1, Fig. 2). This could be attributed to the absence of anthropogenic disturbances, and higher organic matter content in forest soils and vice versa in cultivated soils. These are further consistent with the previous reports (Sofi, Rattan & Datta, 2012; Chemeda, Kibret & Fite, 2017). The mean values of soil pH in the surface (0–30 cm) and sub-surface (30-60 and 60–90 cm) layer varied from 6.13–7.17, 6.37–7.30, and 7.33–7.50, respectively, with minimum values observed in forest soil and maximum in paddy-oilseed soils (Fig. 2). Moreover, an increment was observed along the soil depth, and all the examined soil depths differed significantly (*p* < 0.05) (Table 1, Fig. 2). In general, soils were slightly acidic to slightly alkaline in reaction. This might be ascribed to the absorption of bases by tree biomass, the acidic character of litter following its degradation, the accumulation of organic matter in forest soils, and rapid oxidation owing to tillage practices in cultivated soils. Our results agree with the prior reports (Muche, Addis & Molla, 2015; Kiflu & Beyene, 2013).

**Table 1:** Two-way ANOVA for the effect of land use system (LUS) and soil depth on sand, silt, clay, BD, pH, and N.

**Treatment**	**df**	**Sand**	**Silt**	**Clay**	**BD**	**pH**	**N**
		*p*	*p*	*p*	*p*	*p*	*p*
LUS	4	<0.001	<0.001	<0.001	<0.001	<0.001	<0.001
SD	2	<0.001	<0.001	<0.001	<0.001	<0.001	<0.001
LUS × SD	8	0.02	0.33	0.94	0.99	<0.001	0.06

### Distribution of SOC pools across studied LUSs

Statistical analysis adopting two factor completely randomized design with interaction was employed for the data obtained from investigated LUSs at different depths (Tables 2–6). Table 2 depicts a two-way ANOVA, reflecting the LUS and soil depth influence on TOC. The mean values of TOC under surface (0–30 cm) soils varied from 36.12–64.32 Mg ha^−1^ and in sub-surface (30-60 and 60–90 cm) layers from 27.48–55.66 Mg ha^−1^ and 18.90–44.54 Mg ha^−1^, respectively across studied LUSs. The amount of TOC differed significantly (*p* < 0.05) among studied LUSs with maximum content under forest soils and minimum under paddy-oilseed soils. Furthermore, a significant decline has been observed in TOC content along the soil depth (*p* < 0.05). The increased amounts of TOC in the forest soils might be associated with a significant annual inclusion of organic materials as plant litter that persists in the soil because of no intrusion and seasonal tillage (Smith, 2007; Baker et al., 2007). The rate of decomposition is impeded by the lower temperature conditions in high altitudes which accounts for increased carbon values. The smaller level of TOC in croplands could be because of the negligible surface cover, removal of biomass in harvested products, elimination of crop residue, and increased tillage which enhances the loss of carbon (Smith, 2007). Moreover, increased TOC levels in the upper layer might be attributed to increased quantities of litter inputs in the surface layer. These findings conform with Haynes (2005), Smith (2007), Baker et al. (2007), Kalambukattu et al. (2013), Sofi et al. (2016), Giannetta et al. (2018) and Giannetta et al. (2019).

**Table 2:** Effect of LUSs on TOC (Mg ha^−1^) at different depths.

**Depth/Land use**	**Depth-1**	**Depth-2**	**Depth-3**	**Factor mean**
Forest	64.32 ± 0.63	55.66 ± 0.81	44.54 ± 0.93	54.84^**a**^
Pasture	59.33 ± 0.34	52.58 ± 0.52	42.14 ± 0.57	51.35^**b**^
Apple	48.24 ± 0.21	39.97 ± 0.10	31.00 ± 0.10	39.73^**c**^
Saffron	41.28 ± 0.36	30.62 ± 0.33	21.73 ± 0.48	31.21^**d**^
Paddy-Oil seed	36.12 ± 0.22	27.48 ± 0.28	18.90 ± 0.16	27.50^**e**^
Factor Mean	49.85^**a**^	41.26^**b**^	31.66^**c**^	
C.D (*p* < 0.05)	Land Use = 0.79	Depth = 0.61	Land use × Depth = 1.37	

**Notes.**

Depth-1 = 0–30 cm Depth-2 = 30–60 cm Depth-3 = 60–90 cm

Mean values possessing different letters are significantly different at probability level of *α* 0.05.

Table 3 depicts the mean values of WBC under surface (0–30 cm) soils varied from 11.70–22.00 g kg^−1^ and in sub-surface (30-60 and 60–90 cm) layers from 11.46–18.86 and 10.36–16.36 g kg^−1^, respectively across studied land use systems. The amount of WBC differed significantly (*p* < 0.05) among studied LUSs with maximum content under forest soils and minimum under paddy-oilseed soils, while saffron and paddy-oilseed were at par. Further, a significant (*p* < 0.05) reduction in WBC was recorded along the soil depth. Higher organic carbon in surface soils of forests has also been recorded by Miller & Grardiner (2001), Kaleem & Ghulam (2005), Yimer, Ledin & Abdulakdir (2007), Jamala & Oke (2013) and Yihenew, Fentanesh & Solomon (2015). The low organic carbon in cultivated lands might be due to the rapid mineralization and loss of carbon from soil (Chauhan, Pande & Thakur, 2014), long-term cultivation under submerged conditions resulting in stable aggregate breakdown and SOM degradation (Yang, Yang & Ouyang, 2005), eventually deteriorates soil quality. The reports are further supported by the results of Liding et al. (2011), Mojiri, Aziz & Ramaji (2012), Wiesmeier et al. (2012), Poeplau & Don (2013), Chemeda, Kibret & Fite (2017) and Kaur & Bhat (2017).

**Table 3:** Effect of LUSs on WBC (g kg^−1^) at different depths.

**Depth/Land Use**	**Depth-1**	**Depth-2**	**Depth-3**	**Factor Mean**
Forest	22.00 ± 0.11	18.86 ± 0.52	16.36 ± 0.50	19.07^**a**^
Pasture	16.90 ± 0.52	14.73 ± 0.44	13.33 ± 0.53	14.99^**b**^
Apple	16.86 ± 0.06	13.96 ± 0.87	11.73 ± 0.18	14.18^**c**^
Saffron	12.36 ± 0.06	11.50 ± 0.30	10.80 ± 0.36	11.56^**d**^
Paddy-Oilseed	11.70 ± 0.23	11.46 ± 0.32	10.36 ± 0.24	11.17^**d**^
Factor Mean	15.96^**a**^	14.10^**b**^	12.52^**c**^	
C.D (*p* < 0.05)	Land Use = 0.69	Depth = 0.53	Land Use × Depth = 1.19	

**Notes.**

Depth-1 = 0–30 cm Depth-2 = 30–60 cm Depth-3 = 60–90 cm

Mean values possessing different letters are significantly different at probability level of *α* 0.05.

The mean values of LOC under surface (0–30 cm) soils varied from 2.33–7.20 g kg^−1^ and in sub-surface (30-60 and 60–90 cm) layers from 2.09–4.74 and 1.86–3.28 g kg^−1^, respectively across studied land use systems (Table 4). The amount of LOC differed significantly (*p* < 0.05) among studied LUSs with maximum content under forest soils and minimum under paddy-oilseed soils, following a trend; forest >pasture >apple >saffron >paddy-oilseed. Furthermore, while evaluating the effect of soil depth, a significant decline in LOC content was observed and all depths differed significantly ( *p* < 0.05). The continuous annual inclusion of rapidly decomposable plant litter and increased MBC levels justifies the elevated amount of LOC in forest soils. Whereas the lower values of labile carbon among studied land uses can be attributed to the destabilization of aggregates and accelerated oxidation of SOM in plow and harrow-based traditional cultivation systems (Bayer et al., 2006). These observations are concordant with those of Garten Jr, Post & Hanson (1999), McLauchlan & Hobbie (2004), Cheng, Chen & Luo (2008), and Sofi et al. (2016).

**Table 4:** Effect of LUSs on LOC (g kg^−1^) at different depths.

**Depth/Land Use**	**Depth-1**	**Depth-2**	**Depth-3**	**Factor Mean**
Forest	7.20 ± 0.38	4.74 ± 0.03	3.28 ± 0.02	5.07^**a**^
Pasture	6.82 ± 0.32	4.06 ± 0.18	3.01 ± 0.04	4.62^**b**^
Apple	4.62 ± 0.24	3.87 ± 0.04	2.12 ± 0.04	3.53^**c**^
Saffron	3.66 ± 0.32	3.16 ± 0.16	2.05 ± 0.05	2.95^**d**^
Paddy-Oilseed	2.33 ± 0.07	2.09 ± 0.02	1.86 ± 0.06	2.09^**e**^
Factor Mean	4.92^**a**^	3.58^**b**^	2.46^**c**^	
C.D (*p* < 0.05)	Land Use = 0.30	Depth = 0.23	Land Use × Depth = 0.53	

**Notes.**

Depth-1 = 0–30 cm Depth-2 = 30–60 cm Depth-3 = 60–90 cm

Mean values possessing different letters are significantly different at probability level of *α* 0.05.

**Table 5:** Effect of LUSs on POC (mg kg^−1^) at different depths.

**Depth/Land Use**	**Depth-1**	**Depth-2**	**Depth-3**	**Factor Mean**
Forest	1380.24 ± 60.01	1122.11 ± 15.30	971.11 ± 6.74	1157.82^**a**^
Pasture	1290.34 ± 35.38	1091.36 ± 5.76	894.14 ± 6.48	1091.95^**b**^
Apple	910.52 ± 9.28	788.47 ± 3.68	693.13 ± 3.18	797.37^**c**^
Saffron	870.44 ± 14.84	673.65 ± 7.87	492.42 ± 1.38	678.84^**d**^
Paddy-Oilseed	756.31 ± 19.13	569.12 ± 1.41	390.74 ± 1.37	572.06^**e**^
Factor Mean	1041.57^**a**^	848.94^**b**^	688.30^**c**^	
C.D (*p* < 0.05)	Land use = 33.44	Depth = 25.90	Land Use × Depth = 57.92	

**Notes.**

Depth-1 = 0–30 cm Depth-2 = 30–60 cm Depth-3 = 60–90 cm.

Mean values possessing different letters are significantly different at probability level of *α* 0.05.

**Table 6:** Effect of LUSs on MBC (mg kg^−1^) at different depths.

**Depth/Land Use**	**Depth-1**	**Depth-2**	**Depth-3**	**Factor Mean**
Forest	1458.12 ± 33.51	1232.53 ± 6.08	1004.14 ± 2.76	1232.59^**a**^
Pasture	1370.32 ± 108.48	1189.52 ± 115.43	993.21 ± 102.83	1184.35^**b**^
Apple	1105.16 ± 96.40	933.38 ± 102.01	771.37 ± 90.86	936.64^**c**^
Saffron	968.26 ± 139.23	703.61 ± 130.18	515.30 ± 124.80	729.05^**d**^
Paddy-Oilseed	830.21 ± 141.18	546.38 ± 136.36	365.50 ± 128.10	581.03^**e**^
Factor Mean	1146.42^**a**^	921.28^**b**^	729.90^**c**^	
C.D (*p* < 0.05)	Land Use = 20.23	Depth = 15.67	Land Use × Depth = 35.05	

**Notes.**

Depth-1 = 0–30 cm Depth-2 = 30–60 cm Depth-3 = 60–90 cm

Mean values possessing different letters are significantly different at probability level of *α* 0.05.

**Figure 3:**
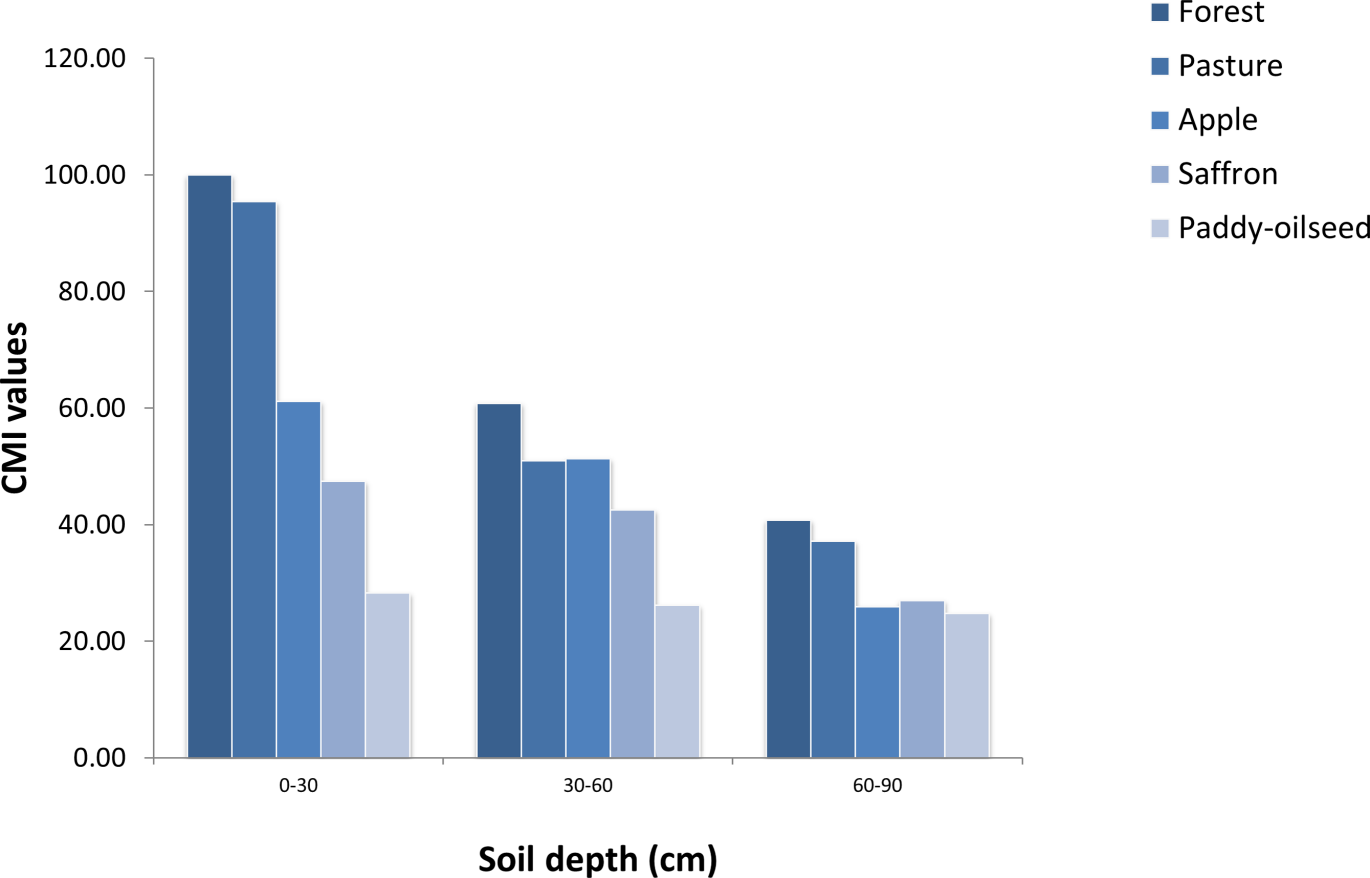
CMI values of different LUSs at varying depths (Reference land use: Forest).

**Figure 4:**
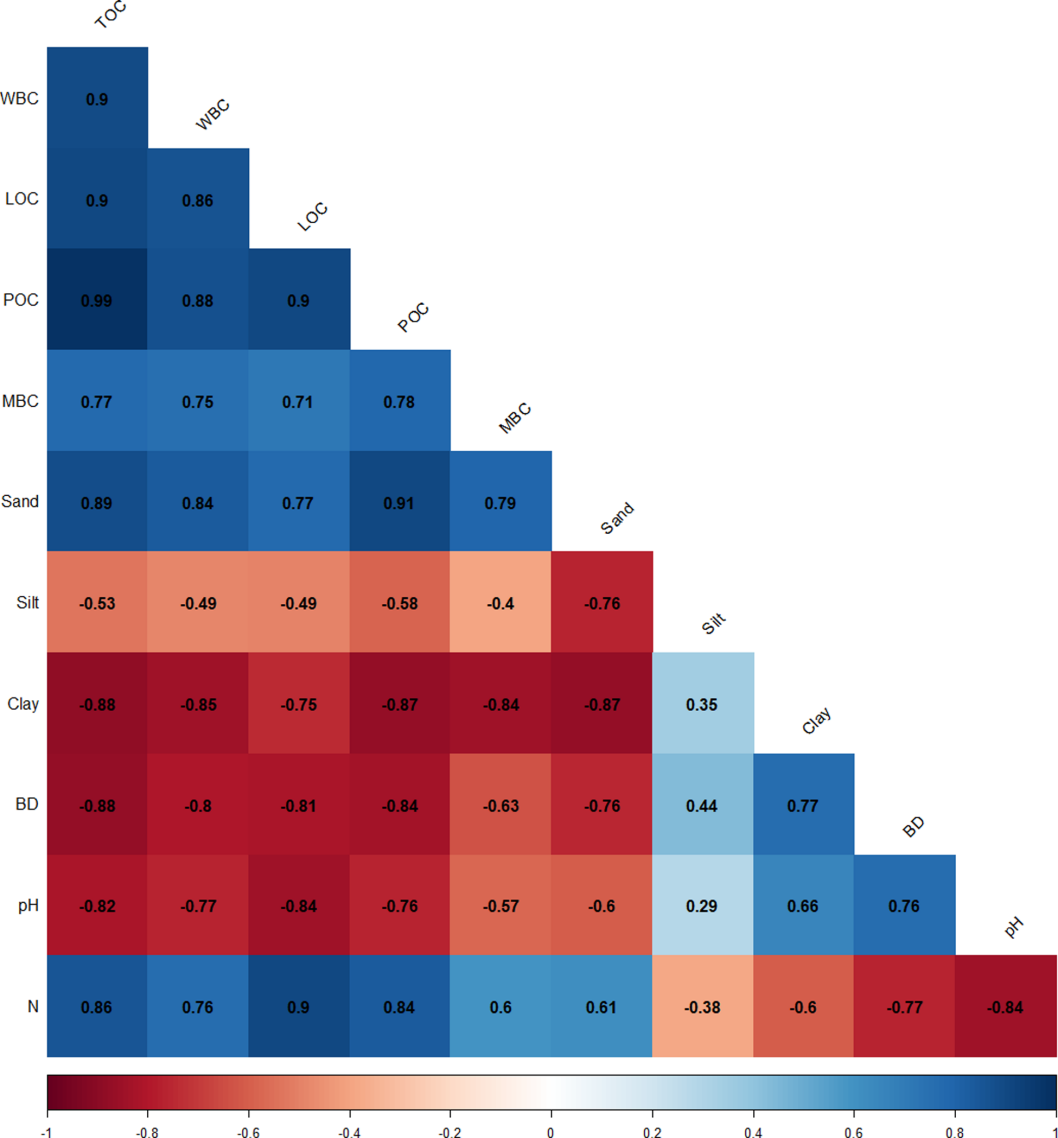
Correlation between physico-chemical characteristics and SOC pools.

The mean values of POC under surface (0–30 cm) soils ranged from 756.31–1,380.24 mg kg^−1^ and in sub-surface (30–60 and 60–90 cm) layers from 569.12–1,122.11 mg kg^−1^ and 390.74–971.11 mg kg^−1^, respectively across studied LUSs (Table 5). The amount of POC differed significantly (*p* < 0.05) among studied LUSs with maximum content under forest soils and minimum under paddy-oilseed soils, following a trend; forest >pasture >apple >saffron >paddy-oilseed. Further, a reduction in POC was recorded along the soil depth and all depths differed significantly (*p* < 0.05). The elevated POC levels in forest soils can be linked to larger litter deposits, which contain additional labile carbon (Laik et al., 2009; Barreto et al., 2011) thus, promote microbial vitality and quantity. In addition, it could also be linked to negligible anthropogenic disturbances, surface cover, and mechanical soil maintenance to decrease erosion. Comparable findings were documented in the central Himalayan range by Kalambukattu et al. (2013), who found that undisturbed land use categories exhibited greater POC owing to carbon buildup that is preserved by soil aggregates. The lowest POC under cultivated soils might be attributed to the deterioration of soil aggregates through tillage that exposes POC to increased breakdown and mineralization resulting in decreased POC levels (Bayer et al., 2006). Furthermore, this fraction does not establish organo-complexes with minerals, thereby making it prone to mineralization (Christensen, 1988). These observations are further in agreement with Six et al. (1998), Figueiredo, Resck & Carneiro (2010), Liu et al. (2014), Sofi et al. (2016), and Poeplau & Don (2013).

The mean values of MBC under surface (0–30 cm) soils ranged from 830.21–1,458.12 mg kg^−1^ and in sub-surface (30–60 and 60–90 cm) layers from 546.38–1,232.53 and 365.50–1,004.14 mg kg^−1^, respectively across studied LUSs (Table 6). The amount of MBC differed significantly (*p* < 0.05) among studied LUSs with maximum content under forest soils and minimum under paddy-oilseed soils. MBC content decreased significantly with soil depth and all depths differed significantly (*p* < 0.05). The size of the microbial reservoir is influenced by LUSs and the management activities of soil. In addition to the above-mentioned reasons, the elevated MBC values recorded under forests could be ascribed to the synthesis of biomass in the rhizosphere and to a lesser extent, reduced soil tillage. The biomass activity of soil might have been enhanced by organic matter inclusion and improved nutrient cycling in forests (Min et al., 2003). The lowest MBC under cultivated soils might be attributed to low vegetation, tillage operations, oxidation of OM and microbial OC declines steadily with a distance from the rhizosphere (Paul & Clark, 1996). These observations are concordant with those of Beare et al. (1994), Purakayastha, Smith & Huggins (2009), Huang & Song (2010), Singh et al. (2011), and Sofi et al. (2016).

**Figure 5:**
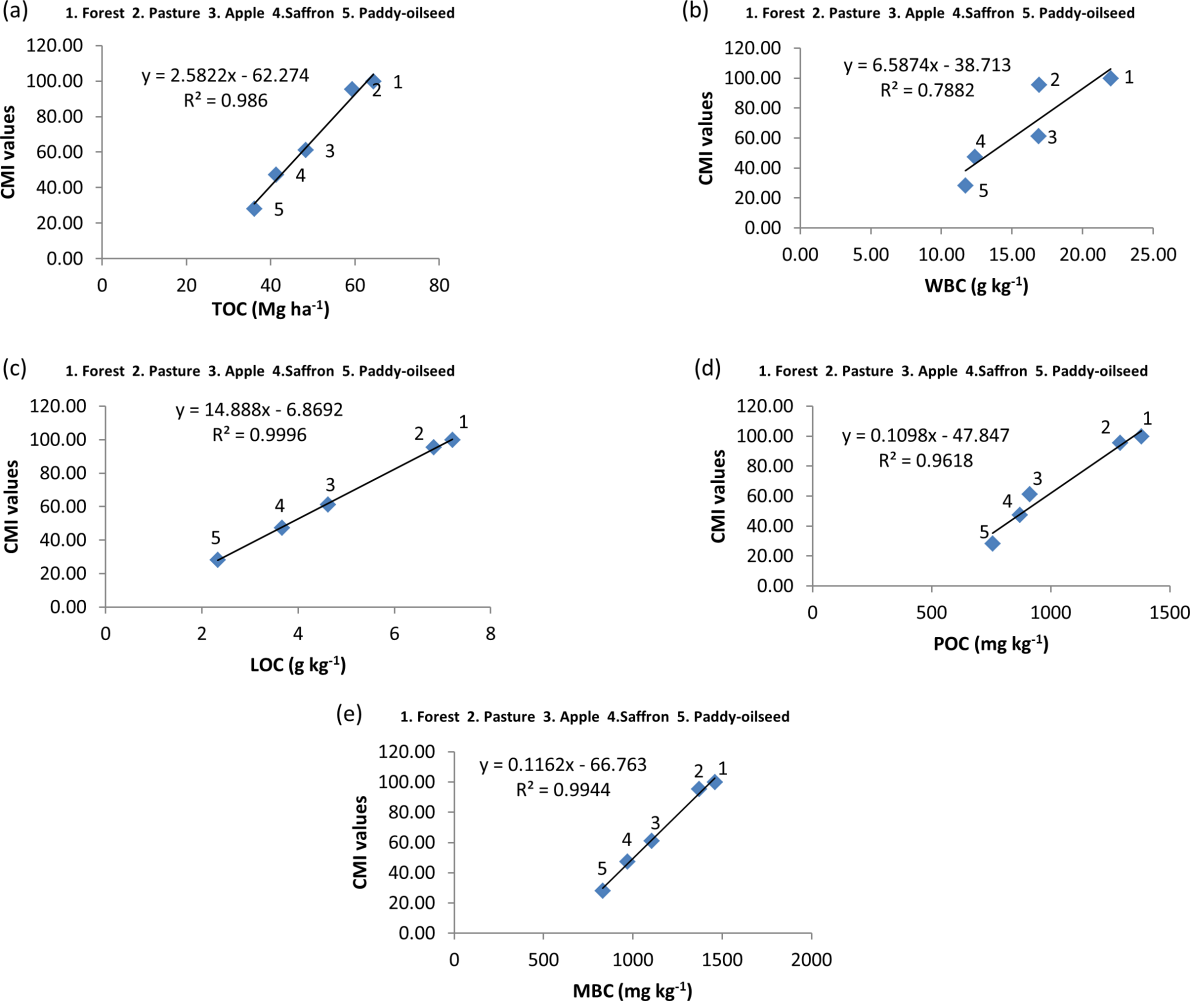
Relationship among SOC pools and CMI at 0–30 cm depth.

**Figure 6:**
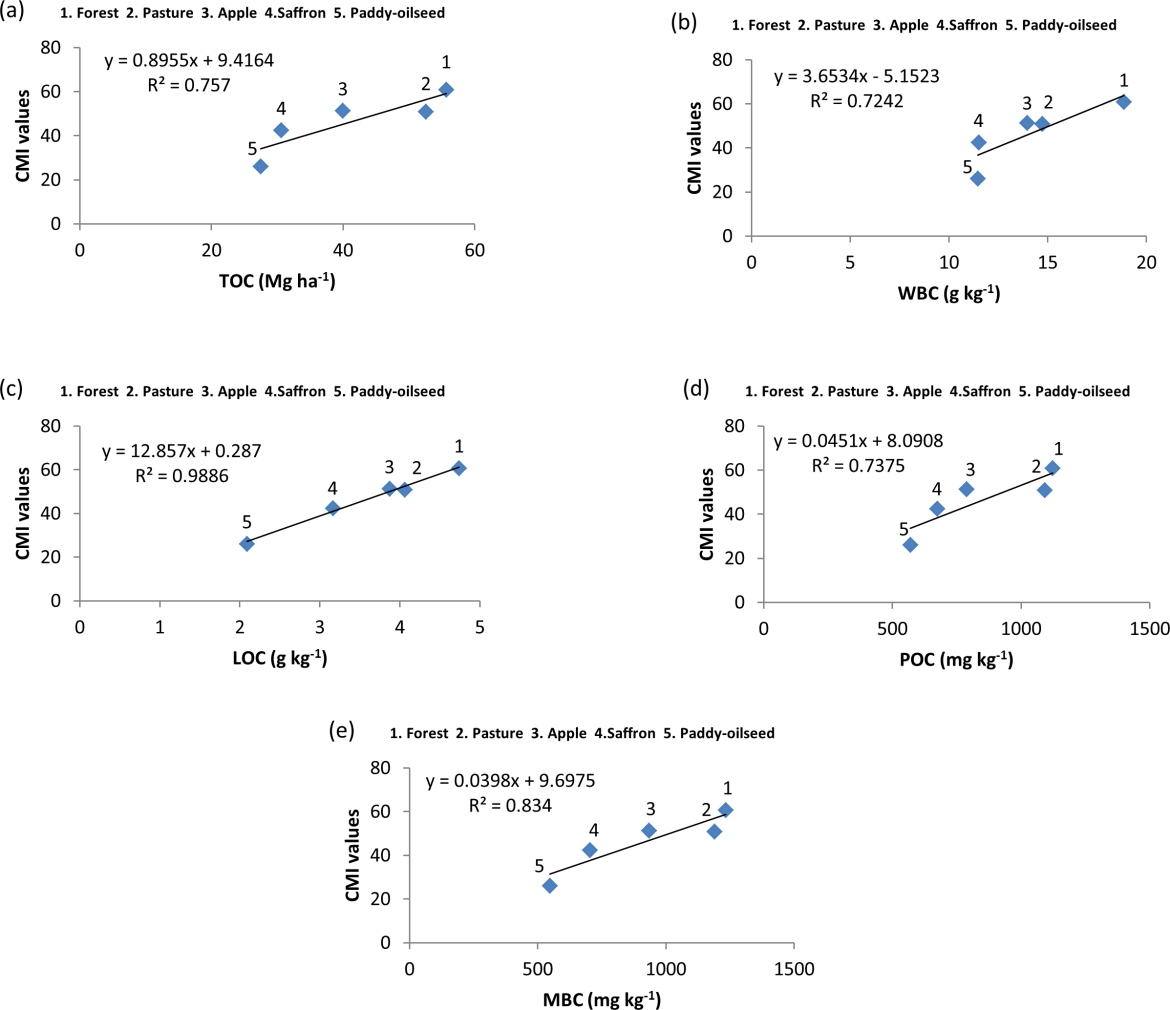
Relationship among SOC pools and CMI at 30–60 cm depth.

**Figure 7:**
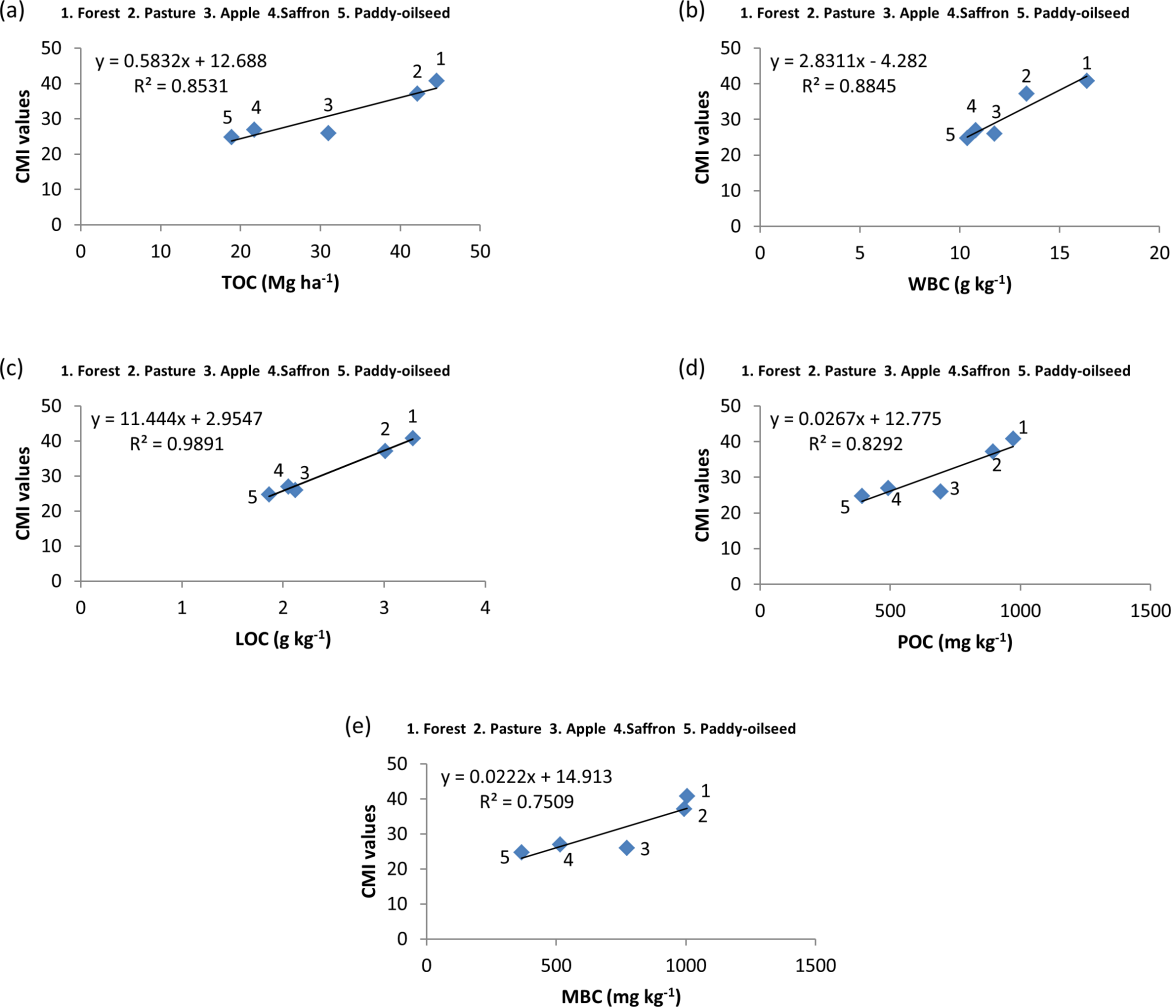
Relationship among SOC pools and CMI at 60–90 cm depth.

### Carbon management index (CMI)

The evaluation of CMI was performed for studied LUSs while taking forest surface soil as a reference for calculation. A significant influence of LUSs and soil depth on CMI values was observed. In this study, the highest CMI values were observed under forest followed by pasture and lowest in paddy-oilseed with the following trend: forest(100) >pasture (95.46) >apple (61.16) >saffron (47.44) >paddy-oilseed (28.29) at surface soils, whereas, CMI showed a significant decline in lower depths across studied land use systems (Fig. 3). This reflects that CMI serves as reliable metrics for estimating variations in SOC pools as well as soil quality. Forest soils had the highest CMI value, which was different from other land uses (Fig. 3), since forest provides a less oxidative environment for the breakdown of POC and POC build-up has been enabled by the prevalence of the thicket awning, a preventive pattern of the macro-aggregates and lesser erodability. These observations are similar to those noticed by Blair, Lefroy & Lisle (1995). The periodic organic matter preservation and reduced depletion concerning forest and pasture have proven to augment the values of CMI (Blair & Crocker, 2000). The lower level of CMI in paddy-oilseed soils indicates that this land use type has lower inputs of carbon and raised turnover levels because of increased temperature and SOC depletion. Similar reports have been noticed by Cao et al. (2013). The usage of nitrogen-based fertilizer has improved biomass thereby aggrandizing SOM. These observations are similar to Vieira et al. (2007) who noticed fertilization and residue inclusion aggrandize the SOM’s lability by 12–46%, thus raising CMI. There exists no definite norm for CMI since it relies on the indigenous LUS of the region; therefore, greater values of CMI signify carbon restoration whereas the least CMI levels denote the depletion of carbon. This is further consistent with reports of Benbi et al. (2015).

### Relationship matrix of physico-chemical characteristics, SOC pools and CMI

A correlation was performed between the physico-chemical characteristics and SOC pools which revealed a positive significant association of N with carbon pools, whereas pH and BD showed a negative significant association with SOC pools (Fig. 4). From regression analysis (Figs. 5, 6 and 7), a positive significant association (high R-squared values) was marked between CMI and different organic carbon pools in surface (0–30 cm) as well as sub-surface (30–60 and 60–90 cm) layers.

## Conclusion

The study concluded that various LUSs and soil depth had an impact on SOC pools and, eventually, the CMI. Soil carbon pool dynamics were better in the case of forest and pasture soils while agricultural perturbations including stubble elimination, tillage, and increased erosion could be a substantial driver of carbon depletion from cultivated soils. However, among cultivated land uses, apple soils showed a better carbon management index. Therefore, land use conversions must be checked to impede carbon depletion. A decrease in organic carbon fractions with change in management practices and soil depth has been observed. Minimizing erosion, the addition of more organic matter, diversifying cropping systems, and minimum or zero tillage operations are important steps in reversing soil depletion and improving soil quality. Therefore, we must adopt best management practices to sequester more carbon which eventually improves the quality of soil and ecological balance and ultimately serve the purpose of global sustainability.

##  Supplemental Information

10.7717/peerj.15266/supp-1 Data S1 Data with replication

## Additional Information and Declarations

### Data Availability

The following information was supplied regarding data availability: The raw data is available in the Supplemental File.

## References

[ref-1] Abad JRS, Khosravi H, Alamdarlou ES (2014). Assessment the effects of land use changes on soil physicochemical properties in Jafarabad of Golestan province, Iran. Bulletin of Environment Pharmacology and Life Sciences.

[ref-2] Addiscott T (1995). Entropy and sustainability. European Journal of Soil Science.

[ref-3] Babu S, Mohapatra KP, Yadav GS, Lal R, Singh R, Avasthe RK, Das A, Chandra P, Gudade BA, Kumar A (2020). Soil carbon dynamics in diverse organic land use systems in north eastern Himalayan ecosystem of India. Catena.

[ref-4] Baker JM, Ochsner TE, Venterea RT, Griffis TJ (2007). Tillage and soil carbon sequestration: What do we really know. Agriculture, Ecosystem and Environment.

[ref-5] Barreto PA, Gama-Rodrigues EF, Gama-Rodrigues AC, Fontes AG, Polidoro JC, Moço MK, Machado RC, Baligar VC (2011). Distribution of oxidizable organic C fractions in soils under cacao agroforestry systems in Southern Bahia, Brazil. Agroforestry Systems.

[ref-6] Bayer C, Martin-Neto L, Mielniczuk J, Pavinato A, Dieckow J (2006). Carbon sequestration in two Brazilian Cerrado soils under no-till. Soil and Tillage Research.

[ref-7] Beare MH, Cabrera ML, Hendrix PF, Coleman DC (1994). Aggregate protected and unprotected organic matter pools in conventional and no tillage soils. Soil Science Society of America Journal.

[ref-8] Benbi DK, Brar K, Toor AS, Singh P (2015). Total and labile pools of soil organic carbon in cultivated and undisturbed soils in northern India. Goederma.

[ref-9] Blair GB, Lefroy DB, Singh BP, Till AR, Cadisch G, Giller KE (1997). Development and use of a carbon management index to monitor changes in soil C pool size and turnover rate. Driven by nature: plant litter quality and decomposition.

[ref-10] Blair GJ, Lefroy RDB, Lisle L (1995). Soil carbon fractions and their degree of oxidation and the development of a carbon management index for agricultural systems. Australian Journal of Agricultural Research.

[ref-11] Blair N, Crocker GJ (2000). Crop rotation effects on soil carbon and physical fertility of two Australian soils. Australian Journal of Soil Research.

[ref-12] Blair N, Faulkner RD, Till AR, Crocker GJ (2006b). Long-term management impacts on soil C, N and physical fertility. Part III. Tamworth crop rotation experiment. Soil and Tillage Research.

[ref-13] Blair N, Faulkner RD, Till AR, Korschens M, Schulz E (2006a). Long-term management impacts on soil C, N and physical fertility. Part II. Bad Lauchstadt static and extreme FYM experiments. Soil and Tillage Research.

[ref-14] Blake GR, Hartge KH (1986). Bulk density. In methods of soil structure and migration of colloidal materials soils. Soil Science Society of America, Proceedings.

[ref-15] Bouyoucos GJ (1962). Hydrometer Method improved for making particle size analysis of soils. Agronomy Journal.

[ref-16] Camberdella CA, Elliott ET (1992). Particulate soil organic matter across grassland cultivation sequence. Soil Science Society of America Journal.

[ref-17] Cao J, Wang X, Sun X, Zhang L, Tian Y (2013). Effects of grazing intensity on soil labile organic carbon fractions in a desert steppe area in Inner Mongolia. Springer Plus.

[ref-18] Chauhan RP, Pande KR, Thakur S (2014). Soil properties affected by land use systems in western Chitwan, Nepal. International Journal of Applied Sciences and Biotechnology.

[ref-19] Chemeda M, Kibret K, Fite T (2017). Influence of different land use types and soil depths on selected soil properties related to soil fertility in Warandhab Area, Horo Guduru Wallaga Zone, Oromiya, Ethiopia. International Journal of Environmental Sciences and Natural Resources.

[ref-20] Cheng X, Chen J, Luo Y (2008). Assessing the effects of short-term Spartina alterniflora on labile and recalcitrant C and N pools by means of soil fractionation and stable C and N isotopes. Geoderma.

[ref-21] Christensen BT (1988). Effects of animal manure and mineral fertilizer on the total carbon and nitrogen contents of soil size fractions. Biology and Fertility of Soils.

[ref-22] Diekow J, Mielniczuk J, Knicker H, Bayer C, Dick DP, Kogel-Knaber I (2005). Carbon and nitrogen stocks in physical fractions of a subtropical Acrisol as influenced by long-term no-till cropping systems and N fertilization. Plant and Soil.

[ref-23] Fayaz A, Shafiq MU, Singh H, Ahmed P (2020). Assessment of spatiotemporal changes in land use/land cover of North Kashmir Himalayas from 1992 to 2018. Modeling Earth Systems and Environment.

[ref-24] Figueiredo CC, Resck VS, Carneiro MAC (2010). Labile and stable fractions of soil organic matter under management systems and native cerrado. Brazilian Journal of Soil Science.

[ref-25] Garten Jr CT, Post WM, Hanson PJ (1999). Forest soil carbon inventories and dynamics along an elevation gradient in the southern. Appalachain Mountains Biogeochemistry.

[ref-26] Giannetta B, Plaza C, Vischetti C, Cotrufo MF, Zaccone C (2018). Distribution and thermal stability of physically and chemically protected organic matter fractions in soils across different ecosystems. Biology and Fertility of Soils.

[ref-27] Giannetta B, Plaza C, Zaccone C, Vischetti C, Rovira P (2019). Ecosystem type effects on the stabilization of organic matter in soils: combining size fractionation with sequential chemical extractions. Geoderma.

[ref-28] Han XZ, Li HB, Horwath WR (2013). Temporal variations in soil CO_2_ efflux under different land use types in the black soil zone of Northeast China. Pedosphere.

[ref-29] Haynes RJ (2005). Labile organic matter fractions as central components of the quality of agricultural soils: an overview. Advances in Agronomy.

[ref-30] Huang J, Song C (2010). Effects of land use on soil water soluble organic C and microbial biomass C concentrations in the Sanjiang Plain in northeast China. Acta Agriculturae Scandinavica, section B-Plant Soil Science.

[ref-31] Jackson ML (1973). Soil chemical analysis.

[ref-32] Jamala GY, Oke DO (2013). Soil organic carbon fractions as affected by land use in the southern guinea sawanna ecosystem of Adamawa state, Nigeria. Journal of Soil Science and Environmental Management.

[ref-33] Jenkinson DS, Powlson DS (1976). The effects of biocidal treatments on metabolism in soil-V: a method for measuring soil biomass. Soil Biology and Biochemistry.

[ref-34] Kalambukattu GJ, Rajdeo S, Ashok KP, Kalaimurthy AK (2013). Soil carbon pools and carbon management index under different land use systems in the Central Himalayan region. Acta Agriculturae Scandinavica, Section B—Soil & Plant Science.

[ref-35] Kaleem A, Ghulam R (2005). Effects of different land use types on soil quality in the hilly area of Rawalkot Azad Jammu and Kashmir. Acta Agriculturae Scandinavica Section B—Soil and Plant.

[ref-36] Kaur R, Bhat ZA (2017). Effect of different agricultural land use systems on physico-chemical properties of soil in sub-mountainous districts of Punjab, north-west India. Journal of Pharmacognosy and Phytochemistry.

[ref-37] Kiflu A, Beyene S (2013). Effects of different land use systems on selected soil properties in south ethopia. Journal of Soil Science and Environmental Management.

[ref-38] Kooch Y, Hosseini SM, Zaccone C, Jalilvand H, Hojjati SM (2012). Soil organic carbon sequestration as affected by afforestation: the Darab Kola forest (North of Iran) case study. Journal of Environmental Monitoring.

[ref-39] Laik R, Kumar K, Das DK, Chaturvedi OP (2009). Labile soil organic matter pools in a calciorthent after 18 years of afforestation by different plantations. Applied Soil Ecology.

[ref-40] Lal R (2001). Potential of desertification control to sequester carbon and mitigate the greenhouse effects. Climatic Change.

[ref-41] Lal R, Kimble JM, Lal R, Follett RF (2002). Why carbon sequestration in soils. Agricultural practices and policies for carbon sequestration in soil.

[ref-42] Lal R (2018). Digging deeper: a holistic perspective of factors affecting soil organic carbon sequestration in agroecosystems. Global Change Boil.

[ref-43] Liding C, Xin Q, Xinyu Z, Qi L, Yanyan Z (2011). Effect of agricultural land use changes on soil nutrient use efficiency in an agricultural area, Beijing, China. China Geographical Science.

[ref-44] Liu MY, Chang QR, Qi YB, Liu J, Chen T (2014). Aggregation and soil organic carbon fraction under different land uses on the tableland of the Loess Plateau of China. Catena.

[ref-45] Luo Z, Wang E, Sun OJ (2010). Soil carbon change and its responses to agricultural practices in Australian agroecosystems: a review and synthesis. Geoderma.

[ref-46] Mahapatra SK, Walia CS, Sidhu GS, Rana KPC, Tarsem L (2000). Characterization and classification of the soils of different physiographic unit in the subhumid ecosystem of Kashmir region. Journal of Indian Society of Soil Science.

[ref-47] Maqbool M, Rasool R, Ramzan S (2017). Soil physico-chemical properties as impacted by different land use systems in district Ganderbal, Jammu and Kashmir: India. International Journal of Chemical Studies.

[ref-48] McLauchlan KK, Hobbie SE (2004). Comparison of labile soil organic matter fractionation techniques. Soil Science Society of America Journal.

[ref-49] Meena VS, Mondal T, Pandey BM, Mukherjee A, Yadav RP, Choudhary M, Singh S, Bisht JK, Pattanayak A (2018). Land use changes: Strategies to improve soil carbon and nitrogen storage pattern in the mid-Himalaya ecosystem, India. Geoderma.

[ref-50] Miller RW, Grardiner DT (2001). Soils in our environment.

[ref-51] Min DH, Islam KR, Vough LR, Weil RR (2003). Dairy manure effects on soil quality properties and carbon sequestration in alfalfa–orchardgrass systems. Communications in Soil Science and Plant Analysis.

[ref-52] Mohamed AE, Natarajan A, Srinivasamurthy CA, Rajendra H, Prakash SS (2017). Assessment of soil quality by using remote sensing and GIS techniques; a case study, Chamrajanagar district, Karnataka, India. Acta Scientific Agriculture.

[ref-53] Mojiri A, Aziz HA, Ramaji A (2012). Potential decline in soil quality attributes as a result of land use change in a hill slope in Lordegan, Western Iran. African Journal of Agricultural Research.

[ref-54] Muche M, Addis KA, Molla E (2015). Assessing the physicochemical properties of soil under different land use types. Journal of Environmental and Analytical Toxicology.

[ref-55] Najar GR, Akhtar F, Singh SR, Wani JA (2009). Characterization and classification of some apple growing soil of Kashmir. Journal of the Indian Society of Soil Science.

[ref-56] Nath AJ, Lal R (2017). Effects of tillage practices and land use management on soil aggregates and soil organic carbon in the north Appalachian region, USA. Pedosphere.

[ref-57] Pal S, Panwar P, Bhardwaj DR (2013). Soil quality under forest compared to other land use in acid soil of North Western Himalaya, India. Annals of Forest Research.

[ref-58] Paul E, Clark F (1996). Soil biology and biochemistry.

[ref-59] Poeplau C, Don A (2013). Sensitivity of soil organic carbon stocks and fractions to different land-use changes across Europe. Geoderma.

[ref-60] Purakayastha TJ, Smith JL, Huggins DR (2009). Microbial biomass an N cycling under native prairie, conservation reserve and no-tillage in Palouse soils. Geoderma.

[ref-61] Sahoo UK, Singh SL, Gogoi A, Kenye A, Sahoo SS (2019). Active and passive soil organic carbon pools as affected by different land use types in Mizoram, Northeast India. PLOS ONE.

[ref-62] Sainepo BM, Gachene CK, Karuma A (2018). Assessment of soil organic carbon fractions and carbon management index under different land use types in Olesharo Catchment, Narok County. Kenya. Carbon Balance Manage.

[ref-63] Singh H, Pathak P, Kumar M, Raghubanshi SA (2011). Carbon sequestration potential of Indo-Gangetic agroecosystem soils. Tropical Ecology.

[ref-64] Six J, Elliott ET, Paustian K, Doran JW (1998). Aggregation and soil organic matter accumulation in cultivated and native grassland soils. Soil Science Society of America Journal.

[ref-65] Smith P (2007). Land use change and soil organic carbon dynamics. Nutrient Cycling in Agroecosystems.

[ref-66] Snyder DJ, Trofymow JA (1984). Rapid accurate wet oxidation diffusion procedure for determining organic and inorganic carbon in plant and soil samples. Communications in Soil Science and Plant Analysis.

[ref-67] Sofi JA, Bhat AG, Kirmai NA, Wani JA, Lone AH, Ganie MA, Dar GIH (2016). Soil quality index as affected by different cropping systems in northwestern Himalayas. Environmental Monitoring and Assessment.

[ref-68] Sofi JA, Rattan KA, Datta SP (2012). Soil organic carbon pools in the apple orchards of Shopian district of Jammu and Kashmir. Journal of Indian society of soil science.

[ref-69] Somasundaram J, Chaudhary RS, Kumar AD, Biswas AK, Sinha NK, Mohanty M (2018). Effect of contrasting tillage and cropping systems on soil aggregation, carbon pools and aggregate-associated carbon in rainfed Vertisols. European Journal of Soil Science.

[ref-70] Subbiah BV, Asija GL (1956). A rapid procedure for the estimation of available nitrogen in soils. Current Science.

[ref-71] Vieira FCB, Bayer C, Zanatta JA, Dieckow J, Mielniczuk J, He ZL (2007). Carbon management index based on physical fractionation of soil organic matter in an Acrisol under long-term no-till cropping systems. Soil and Tillage Research.

[ref-72] Walkley A, Black IA (1934). An examination of the Degtijareff method for determining soil organic matter and a proposed modification of the Chromic acid titration method. Soil Science.

[ref-73] Wiesmeier M, Spörlein P, Geuß UWE, Hangen E, Haug S, Reischl A, Schilling B, Von Lützow M, Kögel-Knabner I (2012). Soil organic carbon stocks in southeast Germany (Bavaria) as affected by land use, soil type and sampling depth. Global Change Biology.

[ref-74] Yadav GS, Das A, Lal R, Babu S, Meena RS, Saha P, Singh R, Datta M (2018). Energy budget and carbon footprint in a no-till and mulch based rice–mustard cropping system. Journal of Cleaner Production.

[ref-75] Yang C, Yang L, Ouyang Z (2005). Organic carbon and its fractions in paddy soils as affected by different nutrient and water regimes. Geoderma.

[ref-76] Yigini Y, Panagos P (2016). Assessment of soil organic carbon stocks under future climate and land cover changes in Europe. Science of the Total Environment.

[ref-77] Yihenew GS, Fentanesh A, Solomon A (2015). Effects of land use types, management practices and slope classes on selected soil physico-chemical properties in Zikre Watershed, North-Western Ethiopia. Environmental Systems Research.

[ref-78] Yimer F, Ledin S, Abdulakdir A (2007). Changes in soil organic carbon and total nitrogen contents in three adjacent land use types in the Bale Mountains, southeastern highlands of Ethiopia. Forest Ecology and Management.

[ref-79] Zhang XW, Han XZ, Yu WT, Wang P, Cheng WX (2017). Priming effects on labile and stable soil organic carbon decomposition: pulse dynamics over two years. PLOS ONE.

